# Age-related alteration of the involvement of CD36 for salivary secretion from the parotid gland in mice

**DOI:** 10.1186/s12576-024-00931-6

**Published:** 2024-07-29

**Authors:** Keitaro Satoh, Yuta Ohno, Haruna Nagase, Masanori Kashimata, Kazunori Adachi

**Affiliations:** 1 Division of Pharmacology, Meikai University School of Dentistry, 1-1 Keyakidai, Sakado, Saitama 350-0283 Japan; 2 Present Address: Division of Pharmacology, Asahi University School of Dentistry, 1851 Hozumi, Mizuho, Gifu, 501-0296 Japan

**Keywords:** CD36, Parotid gland, Salivary gland, Salivary secretion, Aging

## Abstract

This in vivo mouse model study was conducted to investigate the temporal alteration of the function of CD36 in salivary secretion. CD36 was highly expressed in the parotid gland of BALB/c mice. No significant variations were shown in the CD36 levels in the 8-, 48-, and 72-week-old animals. However, pilocarpine-induced salivary secretion was reduced in an age-dependent manner, showing a significantly low level at the age of 72 weeks. Pilocarpine-induced salivary secretion was significantly reduced by pretreatment with a CD36 inhibitor at 8 and 48 weeks, but not at 72 weeks. In senescence-accelerated mice (SAM), the pilocarpine-induced salivary secretion was significantly reduced at the age of 56 weeks, and a significantly lower amount of CD36 was demonstrated in the parotid gland, compared with the control. These results suggest that the involvement of parotid CD36 in mouse salivary secretion is altered with age.

**Supplementary Information:**

The online version contains supplementary material available at 10.1186/s12576-024-00931-6.

## Background

Saliva contributes to mucous membrane protection, self-cleaning, maintaining equilibrium, lubrication, buffering, digestion, and dissolution, all of which play an important role in the preservation of oral health. Thus, the impairment of salivation is associated with numerous oral problems, particularly in the elderly [1]. Any decrease in salivary secretion leads to dryness of the mucosa of the mouth and lips, which further induces some degree of difficulty in pronunciation, mastication, and swallowing, as well as the stabilization of dentures [2]. There may also be associated taste alterations since saliva is the key component in the gustation process [2]. Thus, for the elderly, in particular, it has been thought that maintaining salivation secretion is important to maintain and/or improve the quality of life.

There are many possible causes for decreases in salivary secretion, including aging [1]. According to Thomson [2], salivary secretion is lower in the elderly than in the younger generations. In clinical practice, elderly patients have complained of the decrease in salivary secretion, in both the resting condition and in function (e.g., chewing and/or tasting) [3–7]. Teruya et al. [8] reported that the concentration of salivary adenosine triphosphate (ATP) in the elderly was 1.96 times higher than that of the younger generation, suggesting that there are functional implications between energy synthesis/consumption in the salivary glands and salivary secretion. In addition to ATP, there are other energy sources, such as fatty acids (FAs). In general, the uptake of FAs by cells is important in maintaining cellular homeostasis through ATP production caused by β-oxidation. Currently, CD36 is considered as the most important transporter of FAs [9]. In primary cultures of mouse gingival fibroblasts, Shikama et al. [10] demonstrated that FAs exacerbated periodontitis via *CD36*. This occurs, because CD36 takes up circulating FAs and enhances cellular responses triggered by inflammatory cytokines. Notably, CD36 is a transporter that takes long-chain FAs, such as palmitic acid from the extracellular to the intracellular environment [11]. In the heart, CD36 contributes significantly to energy metabolism [12, 13]. It has been reported that cardiac FA metabolism scintigraphy shows reduced heart uptake of the radiolabeled FA, [^123^I]-β-methyl-P-iodophenyl-pentadecanoic acid (BMIPP), in patients with dilated cardiomyopathy due to CD36 deficiency [14, 15]. In addition, CD36 is expressed on the tongue, and plays a role in some taste reception processes by the uptake of dietary FAs [16]. In bovine mammary epithelial cells, CD36 mediates stearic acid (SA)-induced lipid synthesis, and SA also induces lipid secretion [17]. Thus, CD36 is thought to be involved in the secretory function in the mammary gland. Indeed, in human saliva, larger amounts of FAs originating from the submandibular gland have been observed often, compared with that originating from the parotid gland [18]. However, little is known about the transport of FAs in the salivary glands. In human parotid glands, fat content correlates positively with aging [19]. Therefore, in parotid glands, FA transporters, such as CD36, might be involved in the uptake of FAs and may also participate in cellular responses, such as salivary secretion.

In this study, we used young, middle-aged, and aged BALB/c mice to compare the expression and localization of the CD36 in the three major salivary glands: the parotid, submandibular, and sublingual. The effects of CD36 inhibitors on salivary secretion was investigated using in vivo experiments. In addition, the relationship between CD36 expression, salivary secretion and aging was also investigated in aged individuals of senescence-accelerated mice (SAM strain), in which the lifespan was shortened to approximately 70% [20]. Thus, for the first time, this study showed the importance of the temporal alteration of CD36 in parotid salivary secretion.

## Methods

### Animals

A total of 94 male BALB/c mice at 8 weeks (*n* = 42), 24 weeks (*n* = 17), and 32 weeks (*n* = 35) were purchased from Sankyo Labo Service Corporation, Inc (Tokyo, Japan) and Japan SLC (Hamamatsu, Japan). The 24-week-old mice were bred until 48 weeks, and the 32-week-old mice were bred until 72 weeks and then used in the experiments. A total of 29 male SAM prone 1 (SAMP1) at 8 weeks (*n* = 6), 24 weeks (*n* = 6), and 30 weeks (*n* = 17), and a total of 18 male SAM resistant 1 (SAMR1) at 8 weeks (*n* = 3), 24 weeks (*n* = 3), and 30 weeks (*n* = 12) were purchased from Sankyo Labo Service Corporation, Inc. A total of 30-week-old SAMP1 and SAMR1 were bred until 48 weeks (*n* = 12 and 6, respectively) and 56 weeks (*n* = 5 and 6, respectively) of age and then used in the experiments. The number of animals used in each experiment is summarized in Table 1. The relationship between the actual age and the average life span of the BALB/c mice, the SAMP1 and the SAMR1 were expressed based on previous reports [20, 21] in Supplementary Fig. 1. All of the mice were kept under controlled conditions (23 ± 2 °C, 50% humidity, 12 h light/dark cycle) in the animal facility at Meikai or Asahi Universities. All of the mice were provided with free access to water and standard mice feed (MF; Oriental Yeast Co., Ltd, Tokyo, Japan). The animals were cared for in strict accordance with the Guiding Principles for the Care and Use of Animals in the Field of Physiological Sciences, which has been approved by the Physiological Society of Japan. The Animal Subjects Committee at Meikai University (A2039, B2106, A2206 and A2307) and Asahi University (22–047) approved the experimental protocols.

**Table 1:** Number of animals used in each experiment

Mice	Real time RT-PCR	Western blot analysis	Histological analysis	Measurement of salivary secretion	[^3^H]-palmitic acid uptake assay	Overlapping animals	Actual number of animals
BALB/c	46 (10^a^)	10 (10^a^)	6	32	10	10	94
SAMP1		11 (5^b^)		23 (5^b^)		5	29
SAMR1		9 (6^c^)		15 (6^c^)		6	18

Superscripts indicate that the same animals were used in each experiment

### Measurement of the *CD36* mRNA levels by real-time reverse transcription–polymerase chain reaction (RT–PCR) in the major salivary glands

The BALB/c mice were euthanized using carbon dioxide, and their salivary glands were isolated. The total RNA was then purified using a Tissue Total RNA Purification Mini Kit (Favorgen Ping-Tung, Taiwan), and DNA contamination was eliminated using DNase I (Nippon Gene, Tokyo, Japan). Single-strand cDNA was synthesized from 0.5 μg total RNA by reverse transcription with random and oligo dT primers using the ReverTra Ace qPCR RT Master Mix (Toyobo, Tokyo, Japan). Real-time reverse transcription–polymerase chain reaction (RT–PCR) was performed on 25 μL of the reaction mixture containing each primer, template cDNA, and SYBR Premix Ex Taq II (Takara Bio Inc., Shiga, Japan) using a Thermal Cycler Dice Real Time System (Takara Bio Inc.). Reactions were performed in 40 cycles of 95 °C for 5 s and 60 °C for 30 s after an initial denaturing at 95 °C for 30 s. In addition, melting curve data were obtained by increasing the temperature from 60 to 95 °C. The primer sets were shown as follows: *CD36*: 5#-AGATGACGTGGCAAAGAACAG-3# (forward) and 5#-CCTTGGCTAGATAACGAACTCTG-3# (reverse); and *Gapdh*: 5#-TGTGTCCGTCGTGGATCTGA-3# (forward) and 5#-TTGCTGTTGAAGTCGCAGGAG-3# (reverse). Finally, the gene expression was quantified using a standard curve and normalized to *Gapdh* and the corresponding experimental control. Reactions were run in duplicate.

### Detection of the CD36 protein by Western blot analysis in the parotid gland

Mice were euthanized using an intraperitoneal injection of pentobarbital (100 mg kg^−1^), and their parotid glands were isolated. The glands were homogenized in ice-cold RIPA lysis buffer (Atto Co., Tokyo, Japan) containing protease inhibitors (pepstatin A, aprotinin, and leupeptin), phosphatase inhibitors (NaF, sodium orthovanadate, and sodium glycerophosphate), and 1 mM phenylmethylsulfonyl fluoride. Then, the homogenates were incubated on ice for 15 min and centrifuged at 14,000 × *g* for 10 min. Subsequently, the supernatants were collected, and their protein concentrations were determined using the method established by Bradford [22] using a Bio-Rad protein assay kit (Bio-Rad Laboratories, Hercules, CA). The protein samples were separated by sodium dodecyl-sulfate polyacrylamide gel electrophoresis (SDS–PAGE) using a Mini-Protean 3 Cell system (Bio-Rad). After electrophoresis, the separated proteins were transferred onto a polyvinylidene difluoride filter using a Trans-Blot Turbo System (Bio-Rad). The blots were then blocked at 25 °C for 50 min in skim milk (Morinaga-Nyugyo, Tokyo, Japan) and probed with a primary antibody, rabbit anti-CD36 (GeneTex, Inc., Irvine, CA, USA, #GTX100642; diluted 1:1000), rabbit anti-sodium/potassium ATPase (GeneTex, Inc., #GTX635461; diluted 1:1000), and rabbit anti-α-tubulin antibody (Proteintech, Rosemont, IL, USA, #11224-1-AP; diluted 1:8000) for 120 min. Subsequently, the blots were washed thrice with Tris-buffered saline (pH 7.6) containing 0.05% Tween 20, probed for 90 min with anti-rabbit IgG HRP-linked antibody (Beckman Coulter, Fullerton, CA, USA, #6440-05; diluted 1:10,000), and rewashed. Finally, immunoreactivity was determined using ECL western blotting detection reagents (Cytiva, Tokyo, Japan). Images were acquired using the ChemiDoc MP System (Bio-Rad Laboratories), and the intensity of CD36 was measured with Image Lab 4.1 software (Bio-Rad Laboratories).

### Histological analysis for salivary gland morphology and CD36 protein localization

BALB/c mice were euthanized using an intraperitoneal injection of pentobarbital (100 mg kg^−1^). The parotid glands were then isolated and fixed in 4% paraformaldehyde. Embedding, staining, specimen examination, and virtual slide preparation of histology images were outsourced to Biopathology Institute Co., Ltd (Ooita, Japan). In brief, immunohistochemical and immunofluorescence staining for CD36 was performed on paraffin-embedded sections. The immunohistochemical sections were reacted with a 200-folds dilution of rabbit anti-CD36 antibody (Abcam, Cambridge, UK, #ab133625). The specimens were subsequently treated with N-Histofine^®^ Simple Stain^™^ Mouse MAX PO(R) (Nichirei Biosciences Inc., Tokyo, Japan) and developed with 3,3′-Diaminobenzidine (DAB). The specimens were then nuclear stained with Meyer's hematoxylin. The immunofluorescence sections were reacted with a 500-fold dilution of rabbit anti-CD36 antibody (Abcam, #ab133625), subsequently treated with Alexa Fluor 594 Chicken anti-rabbit IgG (Abcam). Then, the sections were reacted with a 500-fold dilution of Guinea pig anti-Pan-cytokeratin antibody (LifeSpan Biosciences Inc., Lynnwood, WA, #LS-B16812), subsequently treated with Alexa Fluor 488 goat anti-guinea pig IgG (Abcam), and sealed with SlowFade Goldantifade reagent with DAPI (Life Technologies, Carlsbad, CA). The Olympus Net Image Server (Olympus, Tokyo, Japan) was used to observe all of the stained specimens.

### Measurement of cholinergic stimulation-induced salivary secretion

Mice were anesthetized via an intraperitoneal injection of an anesthetic mixture (0.75 mg kg^–1^ medetomidine (Kyoritsu Seiyaku Co., Tokyo, Japan), 4.0 mg kg^−1^ midazolam (Sandoz K.K., Tokyo, Japan) and 5.0 mg kg^−1^ (butorphanol Meiji Seika Pharma Co., Ltd, Tokyo, Japan), at a dose of 0.05 mL per 10 g body weight. The mixture was chosen based on previous reports [23–25]. In addition, saliva volume was determined by a gravimetric method using paper plugs (JM paper point, J. Morita Corp. Osaka, Japan), as performed in our earlier studies [25, 26]. With the animal under anesthesia, pilocarpine (0.5 mg kg^−1^) was injected intraperitoneally. The secreted saliva was then absorbed into paper plugs inserted into the oral cavity and exchanged at 1 min intervals. Subsequently, the saliva-saturated plugs were weighed, and the volume of the secreted saliva was calculated as the difference in the weight of the plug between before and after the saturation. The total saliva volume was calculated by summing the increase in the weight of each paper plug obtained after each 1 min interval from 1 to 20 min after the pilocarpine injection. All saliva volumes were normalized to body weight. For the study of the effects of CD36 on salivary secretion, we used a CD36 inhibitor, sulfosuccinimidyl oleate (SSO). SSO (Cayman, Ann Arbor, MI, USA) was dissolved in dimethylsulfoxide (DMSO; Wako, Osaka, Japan), followed by a 1:200 dilution in saline. The SSO was then administered intraperitoneally at 1.67, 5.04, and 16.8 mg kg^−1^ (dose: 0.035 mL per 10 g body weight) 20 min before administering the pilocarpine. The same volume of 0.5% DMSO was administered to the control group.

### [^3^H]-palmitic acid uptake assay conducted by an automatic sample combustion system in the parotid gland

BALB/c mice were euthanized using carbon dioxide, and their bilateral parotid glands were isolated and placed in 1 mL of PBS at 37 °C. The glands were pretreated with CD36 inhibitor SSO (50 μM) or the same volume of DMSO (control group) for 20 min. The uptake assay was then initiated by adding 50 nCi/10 μL of [^3^H]-palmitic acid (PerkinElmer Inc., Waltham, MA, USA) into the incubation medium. After 20 min, the parotid glands were washed twice in PBS and burned in an automatic sample combustion system (Aloka, Tokyo, Japan). [^3^H] taken up to the parotid glands was collected as water (^3^H–OH), and the β-rays were measured with a liquid scintillation counter LSC-8000 (Aloka). In addition, the decay rate was normalized using gland weight.

### Statistical analysis

**Fig. 1:**
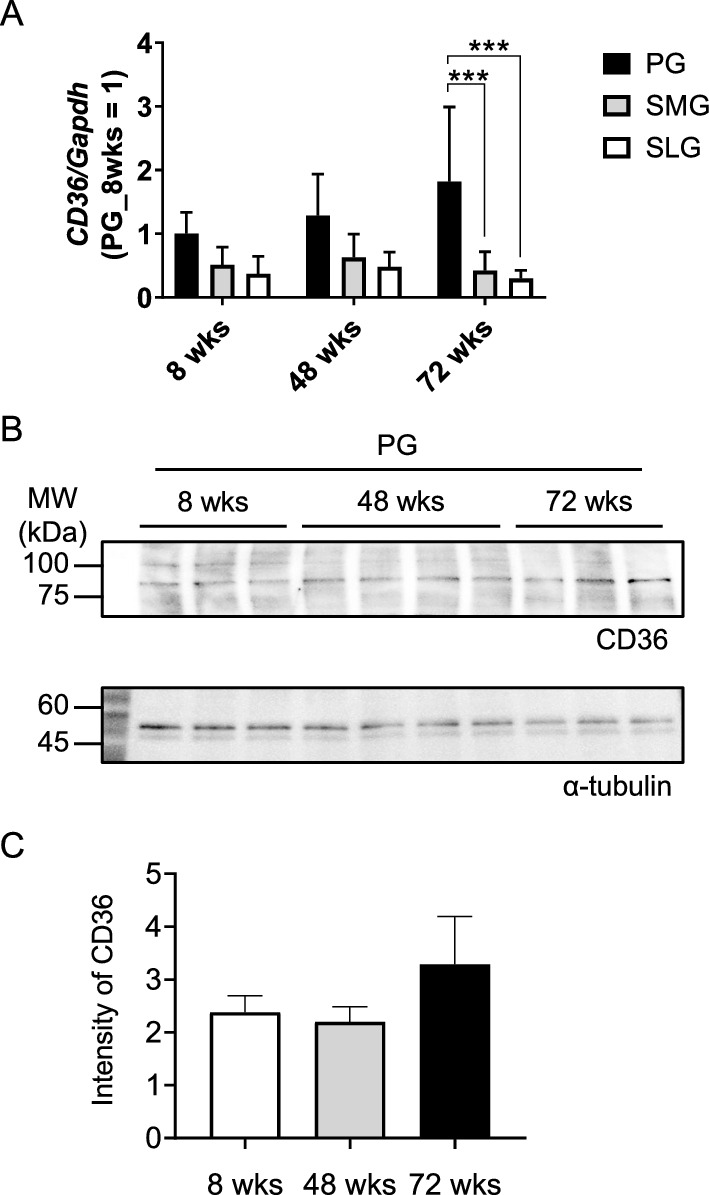
CD36 expression level in the major salivary glands of BALB/c mice aged 8–72 weeks. **A** RNA expression level of *CD36* in the salivary glands of male BALB/c mice aged 8, 48, and 72 weeks (*n* = 18, *n* = 9, and *n* = 19, respectively). The *CD36* expression level was measured using quantitative real-time RT-PCR and normalized to *Gapdh*. Data were comparatively represented by glands. **B** Protein expression level of CD36 in the parotid glands from male BALB/c mice aged 8, 48, and 72 weeks (*n* = 3, *n* = 4, and *n* = 3, respectively). Alpha-tubulin was used as an internal control. **C** Intensity of the immunoreactive bands of CD36. ***p < 0.01 versus the PG group. *PG* parotid gland, *SMG* submandibular gland, *SLG* sublingual gland

In all cases, data were expressed as the mean ± standard deviation (SD) (n = sample size). Statistical comparisons were made using one-way analysis of variance (ANOVA) followed by Tukey’s multiple comparison tests (Figs. 1A, C, 5E, and 6C); two-way ANOVA followed by Dunnett multiple comparisons tests (Fig. 3G–I); one-way ANOVA followed by Dunnett multiple comparisons tests (Fig. 3B); and two-way ANOVA followed by Bonferroni multiple comparisons test (Fig. 5B–D) or unpaired *t* test (Figs. 4, 6B and Supplementary Fig. 2B). A *p* value of < 0.05 was considered statistically significant. These statistical analyses were performed using GraphPad Prism9 (GraphPad Software, La Jolla, CA, USA).

## Results

### Expression and localization of CD36 in salivary glands in BALB/c mice

The *CD36* mRNA level in the salivary glands of BALB/c mice at 8-, 48-, and 72-week-old were compared. The *CD36* mRNA level in the parotid gland was highly observed compared with that in the submandibular and sublingual glands from younger animals, and it reached a significant level at 72 weeks (Fig. 1A). Next, we compared and examined the protein level of CD36 in the parotid gland of BALB/c mice aged 8, 48, and 72 weeks. Western blotting showed that bands that reacted with the anti-CD36 antibody were detected at all ages (Fig. 1B). There was no significant difference in band intensity between 8, 48, and 72 weeks (Fig. 1C).

**Fig. 2:**
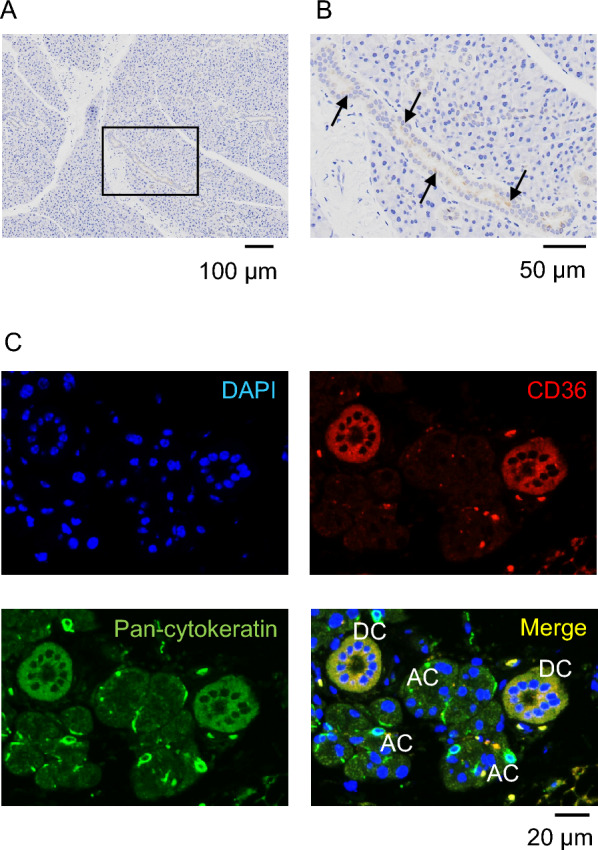
Representative CD36 immunohistochemistry in the parotid gland from BALB/c. **A,** **B** Localization of the CD36 protein in parotid gland from the mice at 8 weeks. The boxes in (**A**) indicated the position of the enraged region in (**B**). The arrows indicated the immunoreaction in the ductal area. **C** Cellular localization of the CD36 protein in the parotid gland in the mice at 72 weeks of age. The blue, red, and green colors show, respectively, the nucleus visualized by the DAPI, the CD36 visualized by the Alexa Fluor 594 Chicken anti-rabbit IgG, and the pan-cytokeratin visualized by the Alexa Fluor 488 goat anti-guinea pig IgG. *AC* acinar cell, *DC* duct cell

The localization of the CD36 protein in the parotid gland of 8-week-old BALB/c mice was examined using immunohistochemistry. Immune responses to anti-CD36 antibodies were detected in the ductal, but not in the acinar area (Fig. 2A, B). A similar localization was observed in 48-week-old animals (data not shown). We also examined the cellular localization of the CD36 protein in the parotid gland using a laser microscopy and immunofluorescence staining. Fluorescence reactive with the anti-pan-cytokeratin antibody, which is designed to target a broad range of cytokeratin isoforms present in various epithelial cells, was detected in both duct and acinar cells. On the other hand, anti-CD36 antibody was detected in the duct, but not acinar cells (Fig. 2C). These results were similar in BALB/c mice at 8 and 48 weeks (data not shown). Taken together, salivary gland CD36 protein was highly expressed in the parotid duct of BALB/c mice regardless of age.

### The effect of the CD36 inhibitor SSO on pilocarpine-induced salivary secretion in BALB/c mice

**Fig. 3:**
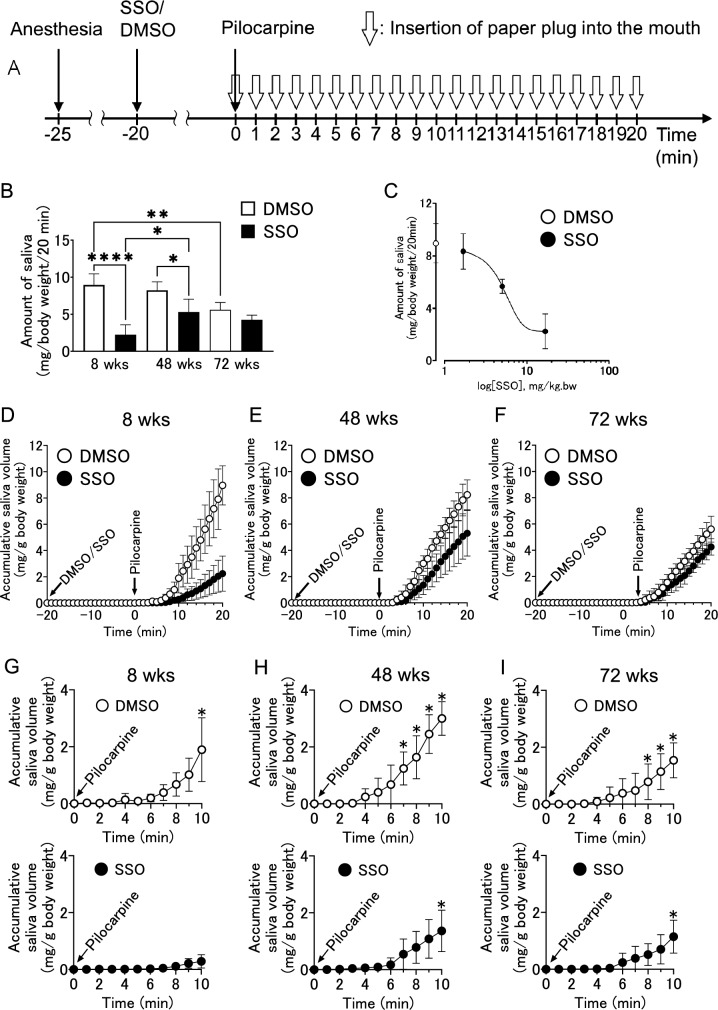
SSO effect on pilocarpine-induced salivary secretion in male BALB/c mice aged 8–72 weeks. **A** Experimental design for measurement of salivary secretion. **B** Total saliva volume induced by pilocarpine treatment and inhibitory effect of SSO in male BALB/c mice aged 8, 48, and 72 weeks. *p < 0.05, **p < 0.01, ****p < 0.001. **C** The dose-dependent inhibitory effect of the SSO (1.68, 5.04, 16.8 mg kg^−1^ intraperitoneal injection) on the pilocarpine-induced salivary secretion. **D**–**F** Accumulative saliva volume within every 1 min interval at each time point after the pilocarpine treatment at ages 8, 48, and 72 weeks, respectively. **G**–**I** Accumulative saliva volume during the first 10 min after pilocarpine treatment were restated separately for the SSO and DMSO groups. *p < 0.05 vs. the treatment at 0 min. All experiments were conducted with four animals in each group

To investigate the role of CD36 in salivary secretion in the parotid gland, the effects of the CD36 inhibitor SSO on pilocarpine-induced salivary secretion were examined in 8-, 48-, and 72-week-old BALB/c mice. Twenty min after administering the SSO or DMSO, pilocarpine was administered to induce salivary secretion, which was measured every minute for 20 min (Fig. 3A). A comparison of the total saliva volume for 20 min after the administration of the pilocarpine revealed that the volume was lower at 72 weeks than at 8 weeks in the DMSO group (Fig. 3B). Similarly, compared with 8-week-old mice in the SSO group, 48-week-old mice secreted a large amount of saliva, but 72-week-old mice did not (Fig. 3B). Regarding the effects of the SSO between the same numbers of weeks old mice, significant differences were observed between the DMSO and SSO groups at 8 and 48 weeks, but not at 72 weeks (Fig. 3B). Figure 3C shows the inhibitory concentration curve of SSO on pilocarpine-induced salivary secretion in 8-week-old BALB/c mice, and it was observed in a dose-dependent manner. In relation to the time course and accumulative saliva volume, the pilocarpine-induced saliva volume tended to be lower in the SSO group than in the DMSO group at 8, 48, and 72 weeks (Fig. 3D, E, and F). In addition, the duration until the pilocarpine-induced salivary secretion reached a significant level (v.s. 0 min: initial secretion rate) was evaluated. In the DMSO group, significant secretion was revealed at 10, 7, and 8 min in mice at 8, 48, and 72 weeks, respectively (Fig. 3G, H, and I, respectively). In the SSO group, significant secretion was revealed at 10 min at both 48 and 72 weeks (Fig. 3H, I, respectively); however, no significant secretion was shown within 10 min at 8 weeks (Fig. 3G).

### The effect of SSO on [^3^H]-palmitic acid uptake in BALB/c mice parotid gland

**Fig. 4:**
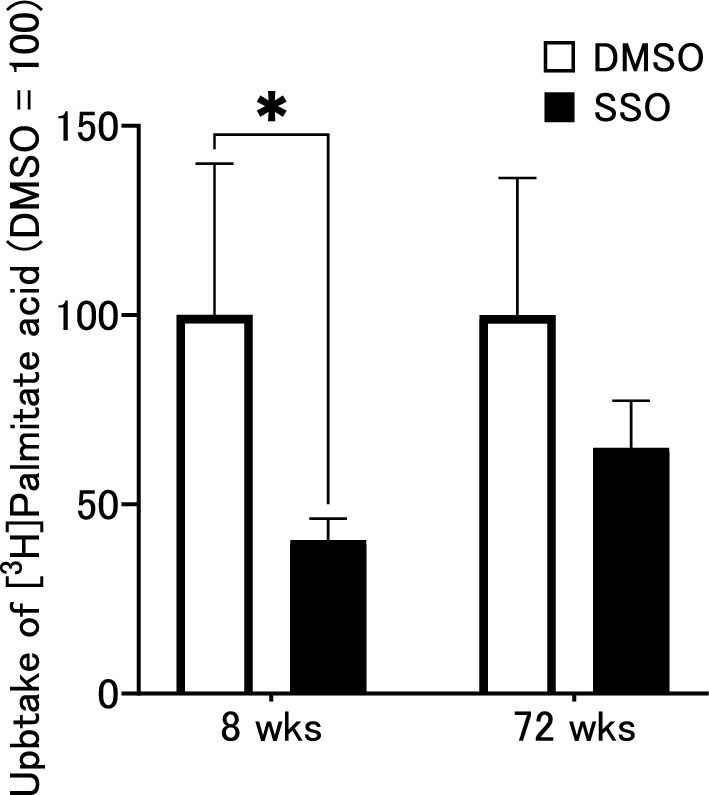
SSO effect on the [^3^H]-palmitate acid uptake in the parotid gland of male BALB/c mice aged 8 and 72 weeks. After pretreatment with SSO or DMSO for 20 min, glands were incubated for 20 min. The uptake of [^3^H] palmitate acid was normalized using gland weight and expressed as 100% of the DMSO group. *p < 0.05. All experiments were conducted with five animals per group

To assess the effects of SSO on FAs uptake in the parotid glands of 8- and 72-week-old BALB/c mice, in vitro [^3^H]-palmitic acid uptake assays were performed. In the 8-week-old mice, the amount of [^3^H] was significantly reduced in the SSO pretreated group (Fig. 4). In contrast, there was no significant decrease at 72 weeks. Thus, SSO clearly inhibited palmitic acid uptake in the parotid gland in the 8-week-old mice.

### Salivary secretion in SAMP1 and SAMR1

**Fig. 5:**
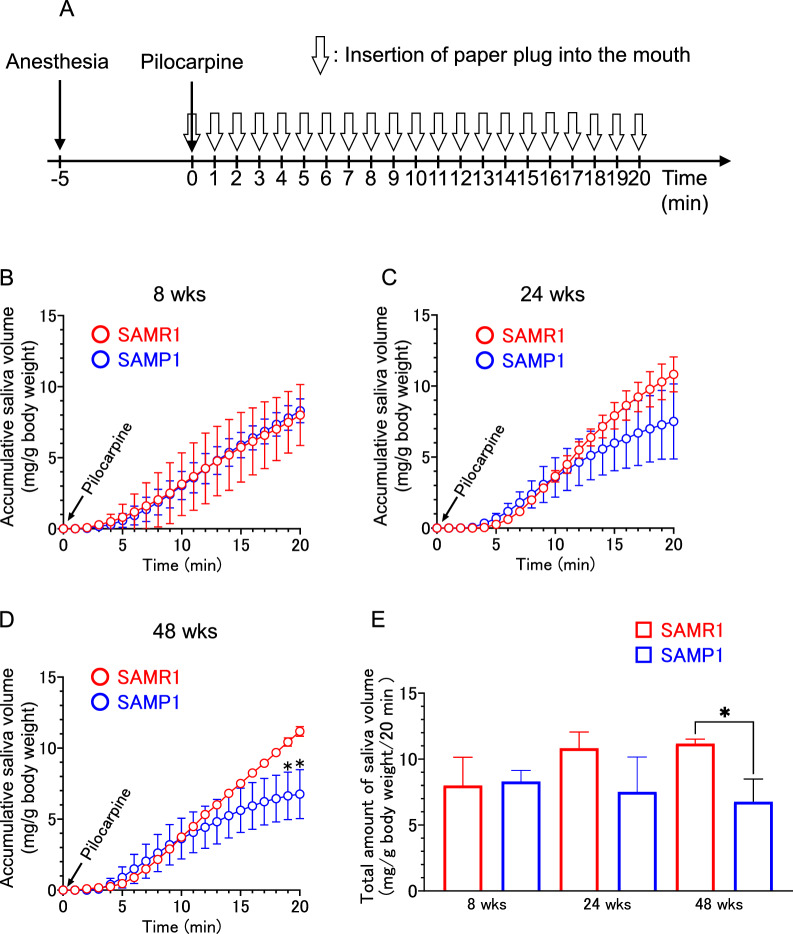
Pilocarpine-induced salivary secretion in male SAMP1 and SAMR1 mice aged 8–48 weeks. **A** Experimental design for measurement of saliva secretion. **B**–**D** Accumulative saliva volume in every 1 min at each time point after pilocarpine treatment at the ages of 8, 24, and 48 weeks, respectively. **E** Total saliva volume after pilocarpine treatment in male SAMP1 and SAMR1 mice aged 8, 24, and 48 weeks. *p < 0.05. All experiments were conducted with three to six animals per group

Next, we examined whether or not the amount of salivary secretion decreases in a senescence-accelerated model. SAMP1 and normal-aged control mice SAMR1 were treated with pilocarpine, and the saliva was collected every minute for 20 min (Fig. 5A). At 8 weeks, no significant difference was observed in salivary secretion between the two strains (Fig. 5B). However, a reduction in salivary secretion in the SAMP1 group was observed from 24 weeks and reached a significant level at 48 weeks (Fig. 5C, D). Comparison of the total saliva volume for 20 min after pilocarpine stimulation revealed that the secretion significantly decreased in the SAMP1 group, compared with the SAMR1 at 48 weeks (Fig. 5E).

### Characterization of the salivary secretion and parotid gland CD36 expression in highly aged SAMP1

**Fig. 6:**
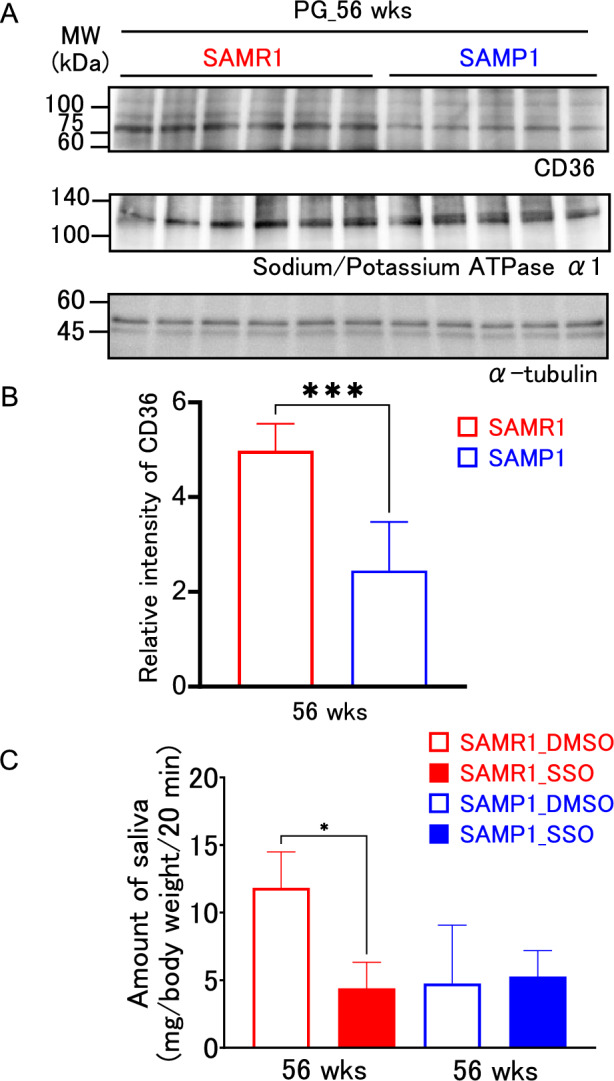
CD36 expression and effect of the SSO on pilocarpine-induced saliva secretion in SAMR1 and SAMP1 mice at 56 weeks of age. **A** Protein expression level of CD36 in the parotid gland from SAMR1 and SAMP1 mice at 56 weeks (*n* = 6 and *n* = 5, respectively). Sodium/potassium ATPase α1 was used as a control for the membrane protein. Alpha-tubulin was used as an internal control. **B** Intensity of the immunoreactive bands of CD36. **C** Total saliva volume induced by pilocarpine treatment and inhibitory effect of the SSO in male SAMR1 and SAMP1 mice aged 56 weeks. *p < 0.05, ***p < 0.001. Except for the SAMP1_DMSO group, the experiments were conducted with three animals in each group. The SAMP1_DMSO group included only two animals

Then, to explore the differences in the highly aged parotid glands of both the SAMP1 and the SAMR1 strains, the CD36 expression at the protein level was examined at 56 weeks. Western blot analysis revealed bands that reacted with the anti-CD36 antibody, in both strains (Fig. 6A). A comparison of the band intensities revealed that SAMP1 had a significantly weaker band intensity, compared with the SAMR1 (Fig. 6B). However, no difference was observed in the bands reacting with antibodies against sodium/potassium ATPase α1, a functional protein present in the cell membrane. We also confirm that this trend was similar at 48 weeks (Supplementary Fig. 2B). Moreover, a comparison of the total saliva volume for 20 min after the administration of pilocarpine revealed that a significant difference was observed between the DMSO and the SSO groups in the SAMR1, but not in the SAMP1 (Fig. 6C).

## Discussion

In the present study, we demonstrated that: 1. In the major salivary glands of BALB/c mice, CD36 was highly expressed in the parotid gland and localized more in the duct than in the acinus. 2. The CD36 inhibitor significantly decreased salivary secretion up to 48 weeks, but not at 72 weeks in the BALB/c mice. 3. In the parotid glands of SAMP1 at the age of 56 weeks, CD36 was poorly expressed, compared with that of the control SAMR1. 4. In 56-week-old SAMR1, but not the SAMP1, the CD36 inhibitor significantly decreased salivary secretion. Consequently, it is likely that CD36 in parotid ducts was highly related to salivary secretion from ‘younger’ to ‘before elder age.’ Moreover, these results suggested that the functional alteration of CD36 in the salivary secretion varies at different life stages.

To investigate CD36 expression in the salivary glands, especially the parotid glands, experiments using BALB/c mice were performed. *CD36* mRNA levels were significantly higher in the parotid gland than in the submandibular and sublingual glands. In contrast, no change was detected at the protein level between the mice at different weeks of age. In the histological analyses, the localization of CD36 in the parotid gland was higher in the duct than in the acinus, suggesting that CD36 might play a role in the parotid gland function in duct cells. In human saliva in the age range of the 20–40 s, the amount of FAs from the submandibular gland origin was observed at a higher level than FAs from the parotid gland origin [18]. The results of that report suggested that there is a difference in the mechanism of FA uptake between the submandibular and parotid glands, which may be due to the difference in the *CD36* mRNA levels.

CD36 is expressed luminally and basolaterally on renal tubular cells and it has been reported that it takes up FAs primarily from urine and the bloodstream [27, 28]. FAs taken up are used for energy metabolism in the cells. Notably, cardiomyocytes and renal tubular cells show a significant energy expenditure [12, 13, 27, 28], requiring β-oxidation from FAs and glycolysis of blood glucose [12]. In the same manner seen in water reabsorption and electrolyte transport in the renal tubules, the salivary gland ducts play an important role in saliva production [29]. Taken together, these results suggested that duct cells expressing CD36 in the parotid gland take up FAs from the bloodstream and primary saliva and use them for energy metabolism. It has previously been reported that spontaneous Ca^2+^ oscillations lead to ATP release and cell swelling without muscarinic agonists in rat parotid duct cells [30]. It has also been reported that muscarinic agonist-induced Ca^2+^ accumulation leads to enlargement of the luminal space due to shrinkage of parotid ductal cells [31]. Therefore, the swelling of parotid ductal cells related to energy metabolism might affect the salivary volume through the width of the waterway.

SSO binds to CD36 in the cell membrane and inhibits the CD36-mediated transport of FAs into rat adipocytes [32]. The dose of SSO, administrated by intraperitoneal injection, required for inhibition of FAs uptake in rat skeletal muscle was reported to be 40 mg kg^−1^ [33]. On the other hand, it has been reported in vitro that SSO not only alters intracellular pH but also nonspecifically modifies proteins other than CD36 [34]. Therefore, to limit as much as possible the effects of SSO other than its effect on CD36, we tried to keep the concentration of SSO action low. In 8-week-old BALB/c mice, for total saliva volume, at the lower dose of 16.8 mg kg^−1^, intraperitoneal SSO induced a significant reduction in this study. Since the inhibitory effect of intraperitoneal SSO was observed in a dose-dependent manner, the importance of CD36 for salivation in younger mice was emphasized. In addition, the onset of salivary secretion was delayed in the SSO group, compared with the DMSO group at 8, 48, and 72 weeks in the BALB/c mice. These findings suggest that CD36 is involved in the uptake of beneficial FAs and salivary secretion in the early stages after pilocarpine stimulation. In 48-week-old BALB/c mice, SSO also reduced the total saliva volume similarly to that seen in the 8-week-old mice. In contrast, in BALB/c mice at 72 weeks, the effect of SSO on the total saliva volume could not be detected. In addition, in an in vitro [^3^H]-palmitic uptake experiment in BALB/c mice, the uptake into the parotid gland was significantly reduced in the group of 8-week-old mice with SSO administered, but not in the 72-week-old mice. This discrepancy leaves open the possibility that FA transporters other than CD36 are upregulated in the parotid gland with age. Notably, CD36 mediates the uptake of palmitic acid by type II pneumocytes from Wistar rats (body weight 100–120 g, at about 5 weeks) which have a function of pulmonary surfactant secretion [35]. This observation strongly supports our results suggesting that CD36 is involved in salivary secretion in 8- and 48-week-old BALB/c mice. In addition, the importance of CD36 in parotid salivary secretion in young mice is also suggested by the fact that the SSO-induced reduction of salivary secretion is more pronounced in 8-week-old BALB/c mice. In this regard, the influence of the acini, as well as the ducts, cannot be ignored. However, the expression of CD36 in the acini has not been confirmed, and since it has also been reported that CD36 may be localized to vascular endothelial cells [36], SSO may decrease the blood flow into the parotid acini due to vasoconstriction, leading to a decrease in salivary secretion. We did not perform tissue staining of the parotid inflow vascular endothelial cells in this study to clarify these issues. Furthermore, since there are no reports on the relationship between CD36 and vasoconstriction or the effects of aging, it is necessary to investigate how the inhibition of the CD36 in the parotid gland leads to decreased salivary secretion in younger mice.

In this study, pretreatment with SSO reduced the salivary secretion in the 56-week-old SAMR1. However, in the 56-week-old SAMP1 mice, SSO did not suppress the salivary secretion. We also showed that the SAMP1 group had lower parotid CD36 protein levels than the SAMR1 group at the age of 48 and 56 weeks. In comparison with the SAMR1, β-oxidation in the liver and the muscle volume of the soleus and the gastrocnemius were significantly reduced in the SAMP1 group at the age of 48 weeks, although that was similarly observed in 8-week-old mice [37]. Miyagi et al. [38] reported that there were no differences in the localization or protein levels of the important factors of salivary secretion, such as NKCC1, AQP5, and TMEM16A, were observed in the immunostained sections of SAMP1 parotid glands in the 16- and 48-week-old mice. Therefore, in the SAMP1 mice, at 56 weeks, the reduced ATP production caused by β-oxidation may be responsible for the decreased salivary secretion, due to less parotid CD36. Further studies should be conducted to measure the amount of ATP in the salivary glands.

The data obtained from three animal strains with different life spans raised the hypothesis for the temporal alteration of CD36 function and expression for the salivary secretion as shown below. The implicated loss of CD36 in the salivary secretion was induced at around 68.4% of the life span (72-week-old BALB/c in this study [21]) without histological alteration in the parotid glands. The malfunction of CD36 was followed by a reduction in the protein level of CD36 in the parotid gland at around 77.9% of the life span (48-week-old SAMP1 in this study [20]) and was maintained subsequently. In addition, these results suggested a relationship between the functional/histological alteration of CD36 and senescence acceleration. Further investigations (e.g., CD36 expression in the parotid gland of very old BALB/c and any other tissues of SAMP1) are required to confirm that hypothesis.

## Conclusions

This study is the first to show that CD36, a FA transporter, is involved in the parotid salivary secretion in BALB/c mice. Moreover, our results suggested an age-related alteration of the importance of parotid CD36 in mouse secretion. Furthermore, while the mechanism by which aging regulates CD36 expression requires further investigation, these results highlighted the involvement of CD36 in parotid salivary secretion.

## Supplementary Information

Supplementary material 1: Fig. 1. Schematic diagram showing the rate of aging in the mice. The degree of aging is shown in relation to the lifespan of the mice that were used in these experiments.

Supplementary material 2: Fig. 2. CD36 expression level in the parotid gland from SAMR1 and SAMP1 at 48 weeks of age. A: The protein expression level of CD36 in the parotid gland from SAMR1 and SAMP1 at 48 weeks (n = 3 and n = 6, respectively). Sodium/potassium ATPase α1 was used as a control for the membrane protein. Alpha-tubulin was used as an internal control. B: The intensity of the immunoreactive bands of CD36. *p < 0.05.

## Data Availability

The data supporting the findings of this study are available from the corresponding author upon reasonable request.

## Abbreviations

ATP: Adenosine triphosphate
DAB: Diaminobenzidine
DMSO: Dimethylsulfoxide
FA: Fatty acid
HE: Hematoxylin and eosin
HRP: Horseradish peroxidase
RT-PCR: Reverse transcription–polymerase chain reaction
SAM: Senescence-accelerated mice
SAMR1: SAM resistant 1
SAMP1: SAM prone 1
SDS–PAGE: Sodium dodecyl-sulfate–polyacrylamide gel electrophoresis
SSO: Sulfosuccinimidyl oleate
