# Correction: Age-related alteration of the involvement of CD36 for salivary secretion from the parotid gland in mice

**DOI:** 10.1186/s12576-024-00945-0

**Published:** 2024-11-27

**Authors:** Keitaro Satoh, Yuta Ohno, Haruna Nagase, Masanori Kashimata, Kazunori Adachi

**Affiliations:** 1 Division of Pharmacology, Meikai University School of Dentistry, 1-1 Keyakidai, Sakado, Saitama 350-0283 Japan; 2 Division of Pharmacology, Asahi University School of Dentistry, 1851 Hozumi, Mizuho, Gifu, 501-0296 Japan

**Correction: The Journal of Physiological Sciences (2024) 74:38** 10.1186/s12576-024-00931-6

Following publication of the original article [1], error was noticed in the Figs. 3 and 6.

In Fig. 3[A], the symbols between −25 and −20 and the symbols between −20 and 0 on the number line are out of place and in 3[D–I], right edge of the graph is cut-off and the circle symbols have become semicircles;

Incorrect Fig. 3[A, D–I]:
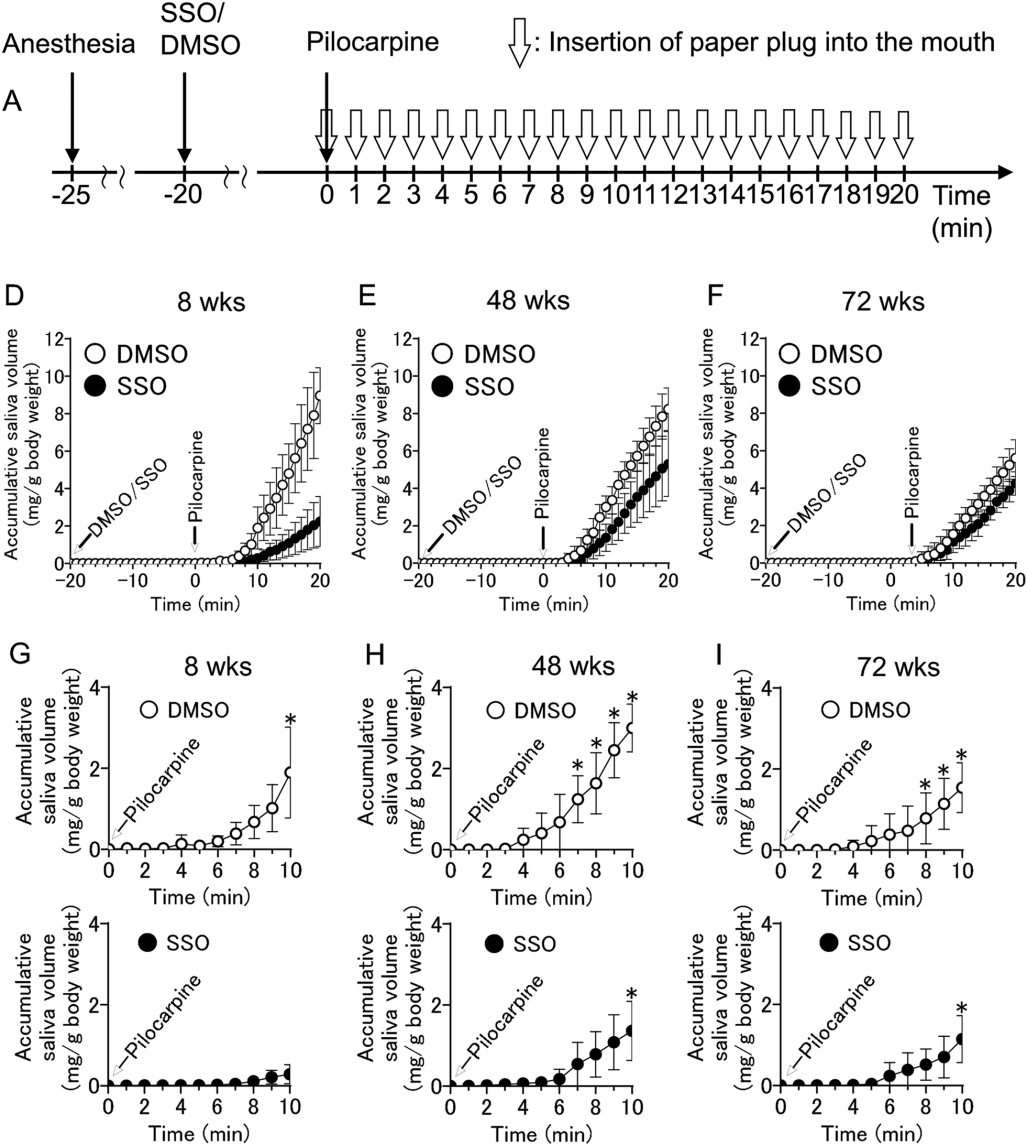


The corrected Fig. 3 should have appeared as shown below:
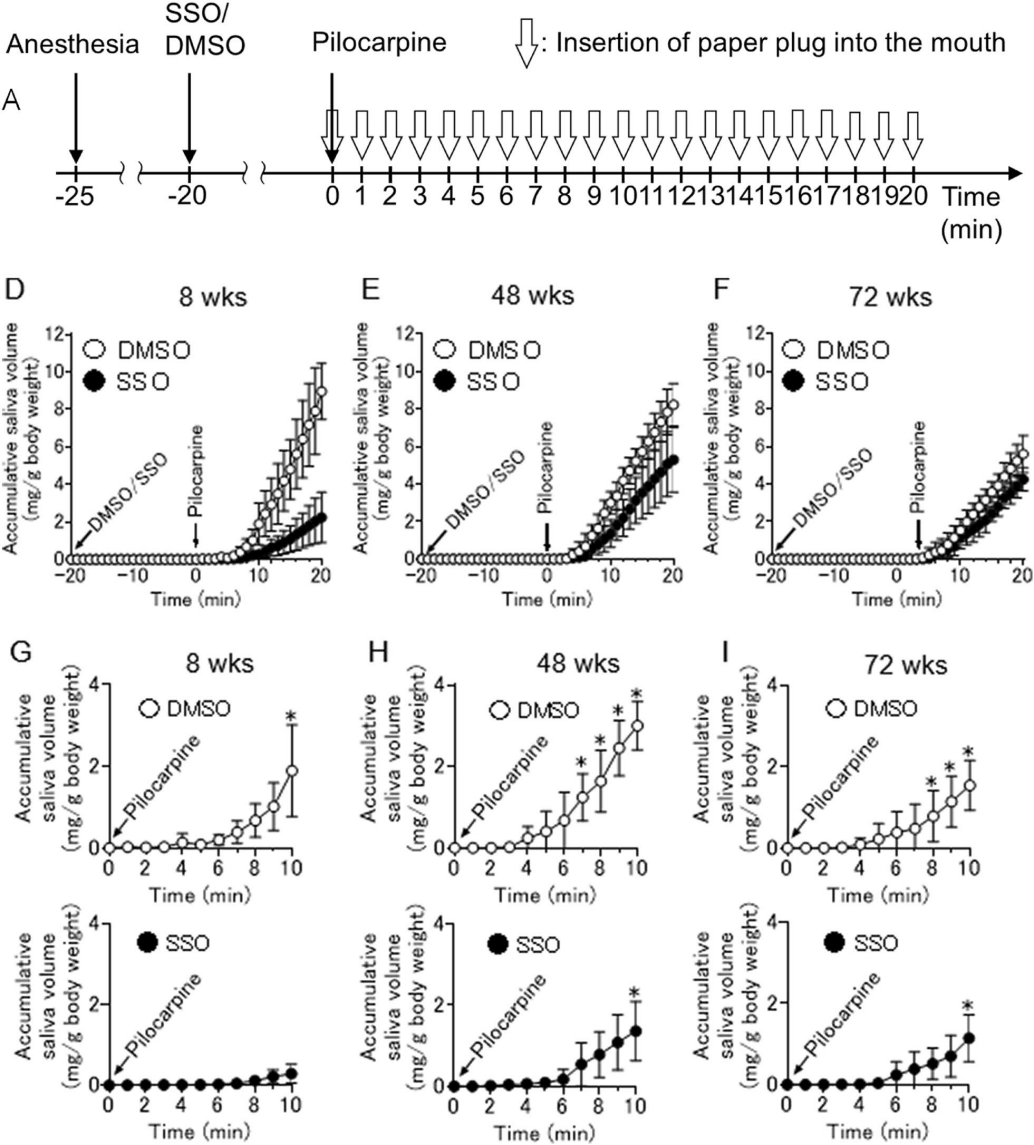


In Fig. 6 [A], “e” and “α” in the display of Sodium/potassium ATPase α1 is overlapped.

Incorrect Fig. 6 [A]
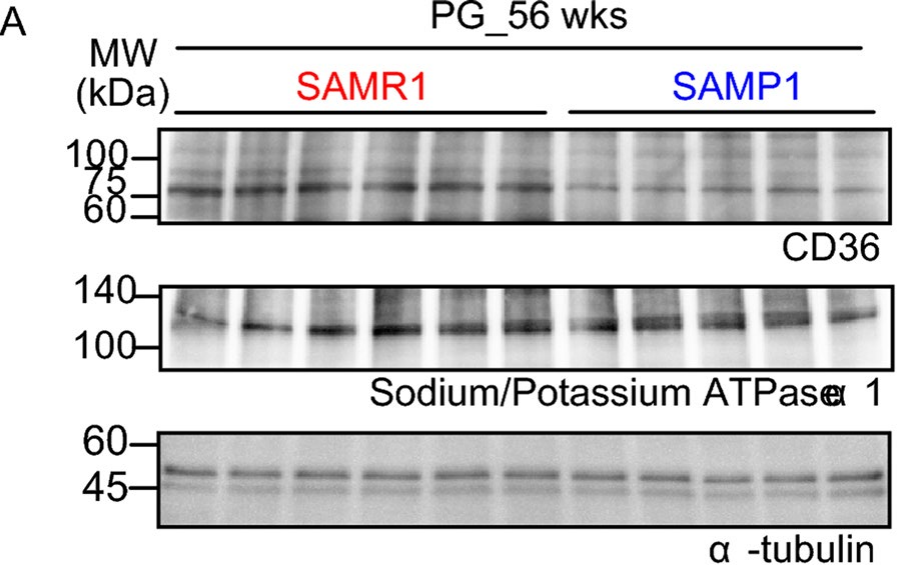


The corrected Fig. 6 should have appeared as shown below:
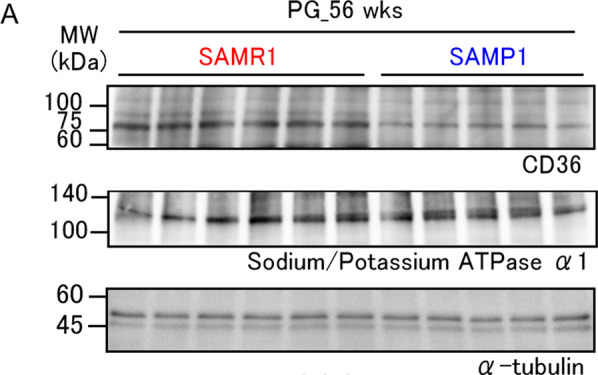


The original article has been corrected.
