# Supplementary figures and images for: Age-related alteration of the involvement of CD36 for salivary secretion from the parotid gland in mice
Source: J Physiol Sci. 2024 Jul 29;74:38. doi: 10.1186/s12576-024-00931-6 (PMC11285320; doi:10.1186/s12576-024-00931-6)

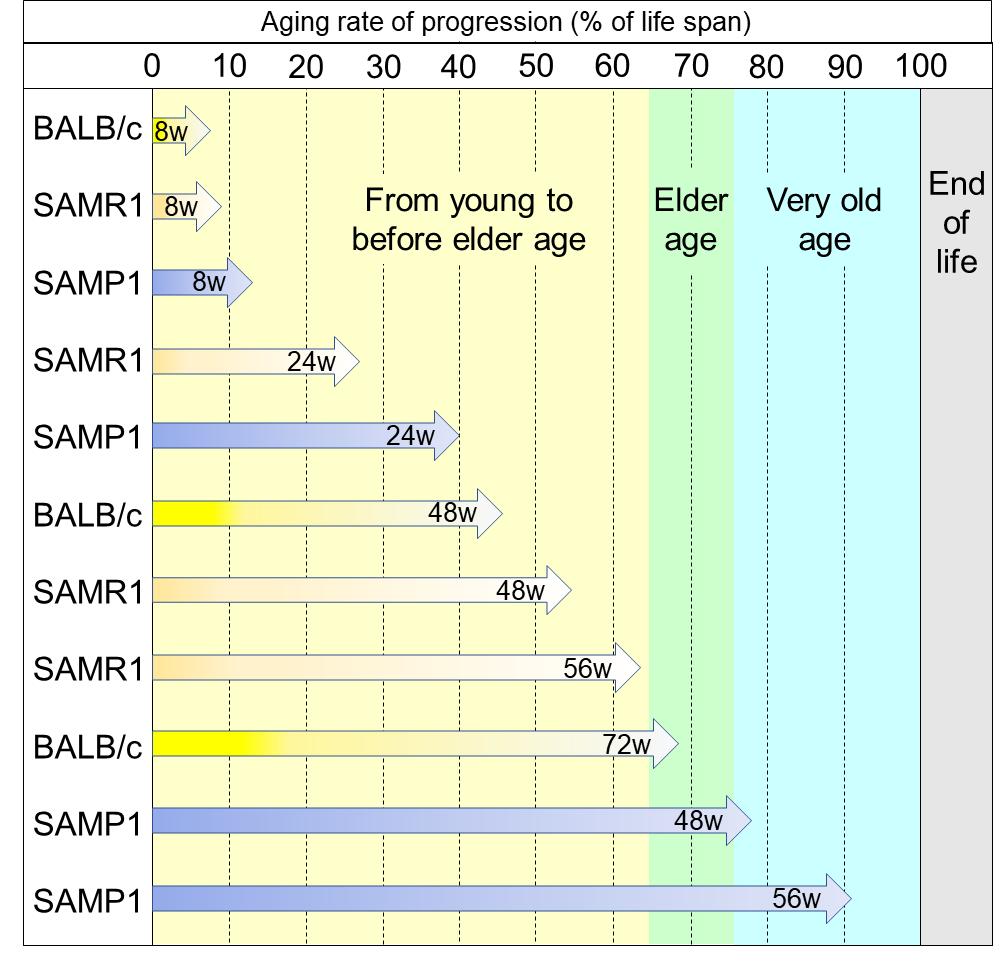

Supplement: Supplementary file 1 — Supplementary material 1: Fig. 1. Schematic diagram showing the rate of aging in the mice. The degree of aging is shown in relation to the lifespan of the mice that were used in these experiments. [file 12576_2024_931_MOESM1_ESM.tif]

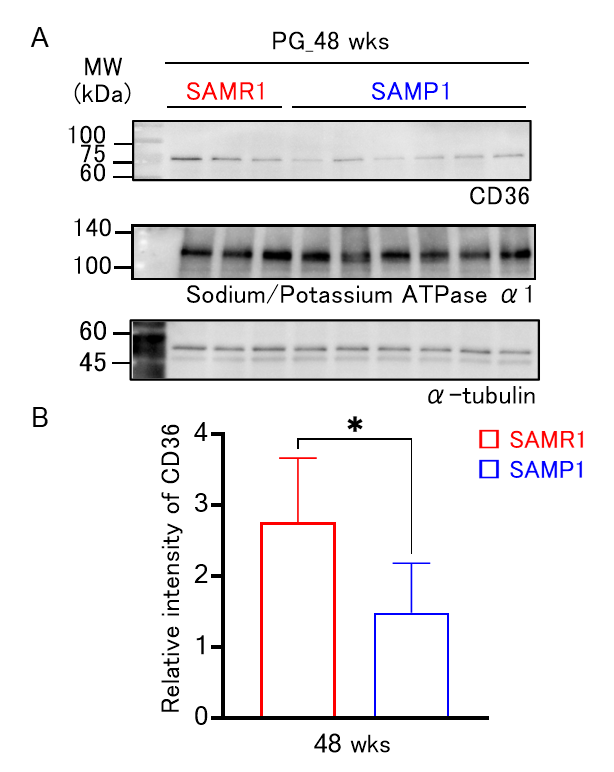

Supplement: Supplementary file 2 — Supplementary material 2: Fig. 2. CD36 expression level in the parotid gland from SAMR1 and SAMP1 at 48 weeks of age. A: The protein expression level of CD36 in the parotid gland from SAMR1 and SAMP1 at 48 weeks (n = 3 and n = 6, respectively). Sodium/potassium ATPase α1 was used as a control for the membrane protein. Alpha-tubulin was used as an internal control. B: The intensity of the immunoreactive bands of CD36. *p < 0.05. [file 12576_2024_931_MOESM2_ESM.tif]
